# Cathepsin K Null Mice Show Reduced Adiposity during the Rapid Accumulation of Fat Stores

**DOI:** 10.1371/journal.pone.0000683

**Published:** 2007-08-01

**Authors:** Marcella Funicello, Michela Novelli, Maurizio Ragni, Teresa Vottari, Cesare Cocuzza, Joaquin Soriano-Lopez, Chiara Chiellini, Federico Boschi, Pasquina Marzola, Pellegrino Masiello, Paul Saftig, Ferruccio Santini, Rene St-Jacques, Sylvie Desmarais, Nicolas Morin, Joseph Mancini, M. David Percival, Aldo Pinchera, Margherita Maffei

**Affiliations:** 1 Dulbecco Telethon Institute at Department of Endocrinology and Metabolism, University Hospital of Pisa, Pisa, Italy; 2 Department of Experimental Pathology, Medical Biotechnologies, Infectivology and Epidemiology, University of Pisa, Pisa, Italy; 3 Department of Morphological-Biomedical Sciences, Human Anatomy and Histology Section, Medical Faculty, University of Verona, Verona, Italy; 4 Biochemical Institute, Christian-Albrechts University, Kiel, Germany; 5 Department of Endocrinology and Metabolism, University Hospital of Pisa, Pisa, Italy; 6 Department of Pharmacology, Merck Frosst Centre for Therapeutic Research, Kirkland, Quebec, Canada; 7 Department of Biochemistry and Molecular Biology, Merck Frosst Centre for Therapeutic Research, Kirkland, Quebec, Canada; University of Parma, Italy

## Abstract

Growing evidences indicate that proteases are implicated in adipogenesis and in the onset of obesity. We previously reported that the cysteine protease cathepsin K (ctsk) is overexpressed in the white adipose tissue (WAT) of obese individuals. We herein characterized the WAT and the metabolic phenotype of ctsk deficient animals (ctsk−/−). When the growth rate of ctsk−/− was compared to that of the wild type animals (WT), we could establish a time window (5–8 weeks of age) within which ctsk−/−display significantly lower body weight and WAT size as compared to WT. Such a difference was not observable in older mice. Upon treatment with high fat diet (HFD) for 12 weeks ctsk−/− gained significantly less weight than WT and showed reduced brown adipose tissue, liver mass and a lower percentage of body fat. Plasma triglycerides, cholesterol and leptin were significantly lower in HFD-fed-ctsk−/− as compared to HFD-fed WT animals. Adipocyte lipolysis rates were increased in both young and HFD-fed-ctsk−/−, as compared to WT. Carnitine palmitoyl transferase-1 activity, was higher in mitochondria isolated from the WAT of HFD treated ctsk−/− as compared to WT. Together, these data indicate that ctsk ablation in mice results in reduced body fat content under conditions requiring a rapid accumulation of fat stores. This observation could be partly explained by an increased release and/or utilization of FFA and by an augmented ratio of lipolysis/lipogenesis. These results also demonstrate that under a HFD, ctsk deficiency confers a partial resistance to the development of dyslipidemia.

## Introduction

Cathepsin K (ctsk) is a member of the papain-like cysteine protease family. Members of this family are generally lysosomal enzymes although some, including ctsk are also found secreted from the cell. Ctsk has potent proteolytic activities against several extracellular matrix components such as collagen I and II, elastase, osteonectin and osteopontin [1]–[3]. Ctsk is most highly expressed in osteoclasts and its major recognized function is in the process of bone remodelling. However, broader potential roles for ctsk have been revealed by the identification of ctsk in a number of other cell and tissue types, including activated macrophages[4], thyroid [5], lung[6], atheroma [7], [8], and skin [9]. Ctsk mRNA and protein were localized in samples of primary breast carcinoma [10] and prostate cancer [11]and it was suggested that this protease, like metalloproteases, may contribute to the invasive potential of certain cancer cells through the digestion of the extracellular matrix.

Several recent reports link cysteine cathepsins with metabolic function. Taleb et al. [12] found that cathepsin S (ctss) gene expression is augmented in adipose tissue of obese humans and defined it as a novel marker of adiposity. Given the involvement of this enzyme in the development of atherosclerotic lesions Rodgers et al. [13], propose that ctss is a molecular link between obesity and atherosclerosis. Inactivation of cathepsin B (ctsb) protects against diet-induced fatty liver disease, as ctsb−/− mice placed on a high carbohydrate diet developed marked obesity, but not the severe hepatomegaly, steatosis and associated dysmetabolic syndrome observed in the wild type (WT) controls placed on the same diet [14].

An even larger body of results indicate that metalloproteases (MMP) also play an important role in obesity and that their activities are important for the early events leading to adipogenesis. Chavey et al. [15] found that MMP-2, MMP-3, MMP-12, MMP-14, MMP-19, and TIMP-1, the endogenous MMP inhibitor, are strongly induced in obese adipose tissues compared with lean tissues. In addition, they and others found that pharmacological inhibition of MMP-2 and MMP-9 decreased adipose conversion *in vivo* [16] and *in vitro* [17], [18]. We have recently reported that ctsk is relatively highly expressed in the white adipose tissue (WAT) and that it is a marker of adiposity as its transcript is induced in murine and human obesity [19]. In addition, we found that ctsk is a novel marker of adipogenesis, since its expression is progressively induced as 3T3-L1 preadipocytes progress to adipose conversion. This may partly account for the increase of ctsk observed in obesity in which *de novo* differentiation takes place. We also demonstrated that the microphthalmia transcription factors (mitf and TFE3), which are known to induce ctsk expression in osteoclasts, are induced in the WAT of obese animals. Interestingly, these transcription factors are activated by macrophage colony stimulating factor (MCSF), which is also induced in the adipose tissue of obese animals [20]. Furthermore, MCSF is able to induce the expression of ctsk in some cell types such as monocytic osteoclast precursors [21]. Ctsk induction therefore appears as a mechanism where different pathways converge, raising the question of the specific role and biological significance this protease may play in adipose tissue and in obesity.

To address this issue, we have employed a loss of function approach and have characterized ctsk null mice from a metabolic perspective. These mice, first described by Saftig [22] and Lazner [23] show impaired resorption of bone matrix and develop osteopetrosis. However, no specific analysis of their metabolism or energy homeostasis had been carried out so far. The results of the present *in vivo* study indicate that ctsk ablation in mice results in an altered body fat content in the young animals and a lowered susceptibility to gain weight on a high fat diet in adult animals. These data, therefore, provides new experimental evidence for the involvement of ctsk in the regulation of fat storage and utilization.

## Results

### Young ctsk−/− mice show reduced fat mass

In previous descriptions of the ctsk null mice (ctsk−/−) [22]–[24] no body weight differences compared to WT mice were presented. Consistent with these findings, we did not observe any significant body weight differences in adult (3 month old and greater) male and female C57Bl6 ctsk−/− mice as compared with C57Bl6 WT animals, when fed a normal chow diet. Dissection of fat pads (adult male epididymal and perirenal and adult female gonadal) showed a moderate (12%), but not significant, reduction in weight for male ctsk−/−, as compared to WT animals. No significant differences between ctsk−/− mice and controls were found for the weights of liver or brown adipose tissue (BAT), or for body length.

**Figure 1:**
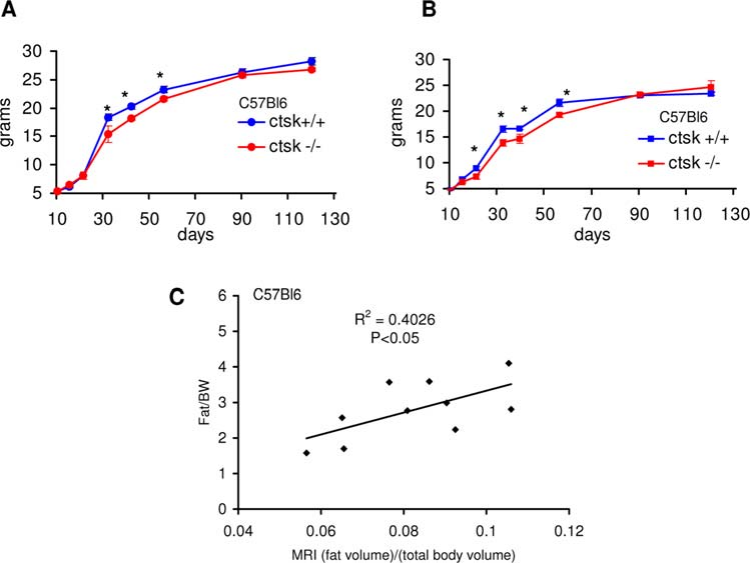
Young C57Bl6 ctsk −/− mice display reduced body weight. Growth curves from 10 to 120 days for male (**A**) and female (**B**) mice (n=12 for males and n=9 for females for each genotype. Statistical details for Bonferroni post‐tests: males df=22, t=2.15 for day 32, t=3.54 for day 42, t=2.08 for day 56; females df=16, t=2.63 for day 21, t=2.81 for day 32, t=2.14 for day 39, t=3.22 for day 56. * P<0.05 . (**C)** Scattergram showing the positive and significant correlation (Spearman regression analysis) (P<0.05) between MRI data, expressed as (fat volume)/(total body volume), and the ratio fat pad/body weight of 10 animals.

However, we noticed that at a younger age, C57Bl6 ctsk−/− mice appeared to have a lower body weight, but similar body length to C57Bl6 WT animals. We therefore measured the growth rate of the animals starting from postnatal day (P) 10 to P120: curves for males and females are respectively shown in Figure 1A and 1B. 2-way analysis of variance (ANOVA) revealed a significant (P<0.01) overall effect of genotype on the body weight during the time window analyzed (F = 9.81, df = 1, F = 8.15 for males and females respectively). A significant interaction between time and genotype was observed for females (F = 2.13, df = 7, time X genotype, P<0.05), but not for males. When Bonferroni post-tests are performed, no significant differences were detectable during the first 2 weeks of life for both genders, whereas at day 21 for females and day 32 for males, ctsk−/− mice show a significantly lower body weight as compared to WT controls. Such a reduction was observed until 8–9 weeks of age, after which no significant differences between the 2 genotypes was observed (see legend of figure 1 for statistical details).

We then investigated the reason for this reduced body weight in the young, by assessing fat mass in 5–8 week-old mice. At this age, the WAT is small, but well defined and dissectible. The weight of the perigonadal fat pad, the most developed fat depot at this age, was significantly decreased in male and female ctsk−/− animals, compared to WT mice (86±7 mg versus 127±9 mg, t = 3.68, df = 58 P<0.001; 35±5 mg versus 56±5 mg, t = 2.77, df = 19, P<0.02 for males and females respectively). No significant differences were found in the weights of liver or BAT. In a group of young C57Bl6 ctsk−/− and ctsk +/+ female mice, adiposity was also assessed non-invasively by magnetic resonance imaging (MRI), that showed a significant reduction (more than 20%) of body fat content in the ctsk−/− mice (t = 2.38, df = 7, P<0.05). Spearman linear regression analysis applied to adiposity (assessed by MRI and expressed as (fat volume)/ (total body volume) against fat pad/body weight in individual animals showed a significant and positive correlation (R = 0.63, df = 9, P<0.05, figure 1C). Based on this result we used the weight of one or more fat pads (if dissectible) as an indicator of adiposity when MRI was not available.

Differences in food intake could account for the reduction in fat depots in young ctsk−/− mice. When this parameter was evaluated, no significant differences in the weight of chow food per mouse per day were found between ctsk−/− and WT mice for young (3.83±1.13 versus 3.95±1.36 g/ mouse/day) or adult animals (4.04±1.35 versus 3.97±1.57 g/ mouse/day).

### Plasma biochemistry of ctsk−/− mice

Since age appeared to determine the extent of differences between C57Bl6 ctsk−/− and WT animals, metabolic parameters (Table 1) were measured using 5–8 week old male (defined as young) and 12–20 week old male mice (defined as adult). 2-way ANOVA was applied to the data reported in table 1. Using this test no significant interaction between genotype and age was assessed for any of the variables under study. When a significant overall effect of genotype was detected a Bonferroni post-test was applied for the pairwise comparison (e.g. young wt versus young KO) and the relative significance is reported in table 1. Non-fasting plasma glucose levels, as well as free fatty acids levels were not significantly altered by the absence of ctsk in young and adult male ctsk−/− mice. However when circulating FFA were measured in a small group of young animals (n = 4) after an overnight fast, we found significantly higher values in ctsk−/− mice as compared to WT controls (845.1±23.2 µEq/l versus 582.6±21.5 µEq/l, t = 7.7, df = 6, P< 0.001). 2-way ANOVA indicates a significant (P<0.05) overall effect of genotype on non-fasting plasma triglyceride levels, with both young and adult ctsk−/− mice showing moderately and not significantly reduced levels. An overall significant reduction (F = 12.11, df = 1 P<0.01) of non-fasting insulin due to ctsk deficiency was also evidenced by 2-way ANOVA and Bonferroni post-test revealed significantly lower levels in adult ctsk−/− mice (t = 3.03, df = 15, P<0.05). An intraperitoneal glucose tolerance test (IpGTT) performed in adult male animals showed that in ctsk−/− mice, postloading plasma glucose levels, as evaluated by the incremental area over basal values during 120 min (ΔG), were significantly (t = 4.59, df = 14, P<0.01) lower than in WT controls (ΔG = 553.74±73.54 and 1145.78±111.82 mg/l/min in ctsk−/− and WT mice, respectively). The glucose stimulated rise in circulating insulin was substantially similar in the two groups. These results indicate that glucose tolerance is improved and insulin sensitivity is well preserved in ctsk-deficient animals. 2-way ANOVA indicates that ctsk deficiency results in a significant overall reduction of plasma leptin (F = 9.08, df = 1, P<0.01). A trend toward reduction of plasma leptin was observed in young male ctsk−/− mice (table 1), and plasma leptin levels were significantly lower in adult male ctsk−/− mice (table 1), as compared with relative controls (t = 3.02, df = 14, P<0.05). Since circulating leptin is a sensitive marker of adiposity, this decrease is consistent with the observed reduction of the fat mass in young ctsk−/− animals and suggests a latent alteration in adiposity in the adult animals. Together these data suggest that the metabolic profile of ctsk−/− mice is somewhat altered in adult animals, where an apparent decrease of adiposity is not observable.

**Table 1:** Metabolic parameters of young and adult C57Bl6 ctsk +/+ and ctsk−/− male mice.

	Young wt	Young ctsk−/−	Adult wt	Adult ctsk−/−
Non fasting glucose (mM)	7.70±0.59 (n = 9)	7.70±0.86 (n = 7)	8.91±0.33 (n = 16)	9.43±0.30 (n = 14)
Non fasting FFA (μEq/l)	620.9±85.3 (n = 9)	515.9±114.2 (n = 8)	326.9±19.1 (n = 12)	383.1±22.2 (n = 9)
Non fasting triglycerides (mM)	1.26±0.15 (n = 9)	1.02±0.16 (n = 6)	1.1±0.07 (n = 16)	0.9±0.05 (n = 14)
Non fasting insulin (ng/ml)	0.9±0.11 (n = 8)	0.65±0.12 (n = 5)	0.65±0.06 (n = 9)	0.29±0.018 (n = 8) **
Non fasting leptin (ng/ml)	3.58±0.31 (n = 10)	3.06±0.18 (n = 8)	5.89±0.34(n = 8)	4.53±0.36 (n = 8) *

Bonferroni post-test

* P<0.05

** P<0.001

### Ctsk−/− mice are partially resistant to high-fat diet-induced obesity

**Figure 2:**
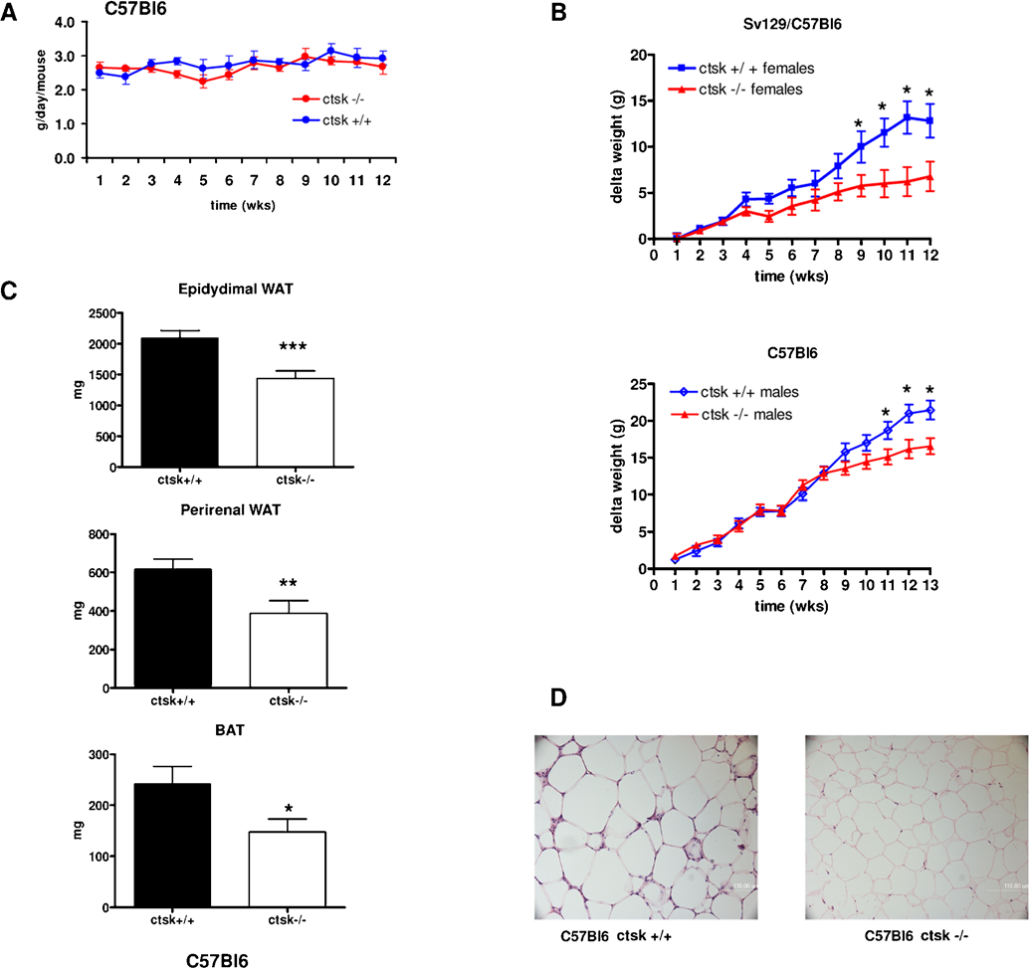
WT and ctsk −/− mice belonging to 2 independent colonies of mice (respectively bred on the mixed sv129/C57Bl6 and pure C57Bl6 genetic background) were fed a high‐fat diet (HFD) starting at 8–9 weeks of age. (A) Representative graph showing food intake in C57Bl6 male mice (n=10). Food intake was monitored weekly in the two groups studied (see text for further explanations) and no significant differences between genotypes were observed. **(B)** Graphs showing the increase in body weight with time for the sv129/C57Bl6 females and for the C57Bl6 males (n=10 for both groups). The increases in body weight were calculated based on the initial body weight at day 0 of HFD feeding. **(C)** Weights of epididymal WAT, perirenal WAT and BAT, taken from 5 month C67Bl6 old male mice (n=9 for ctsk−/− and for WT) after 12 weeks of HFD. Results are mean ± SEM * p<0.05, ** p<0.01, ***p<0.001. **(D)** Representative histological sections of epididymal WAT from C57Bl6 ctsk+/+ (left panel) and ctsk−/− (right panel) mice maintained on a high fat diet for 12 weeks.

Since an altered metabolic phenotype in ctsk−/− mice on a regular diet was clearly observed only in young animals, we challenged adult mice with a high fat diet (HFD) containing 35% fat and 35% carbohydrate (by weight) for 12 (sv129Bl6) or 13 (C57Bl6) weeks to stimulate weight gain. This experiment was performed in the colony of C57Bl6 (ctsk−/− and +/+) mice where the characterization of the adult and young mice so far described had been performed, and in an independent colony bred on the mixed genetic background sv129/C57Bl6 (males and females ctsk−/− and +/+). Results were qualitatively overlapping and the data sets for both groups are summarized in table 2. Due to the important effect exerted by strain on the different variables, data were analyzed only within the separate groups (e.g C57 Bl6 male ctsk−/− versus WT). In the case of sv129/Bl6 mice, males and females were investigated in independent experiments, making any statistical comparison unlikely. For all groups, the intake of the HFD was not significantly different (assessed by 2-way ANOVA) in WT and in ctsk−/− animals. The representative graph shown in figure 2A is relative to weekly measurements of food intake in HFD treated C57Bl6 male mice: the average consumption calculated for these animals is 2.79±0.05 g/mouse/day for WT and 2.69±0.04 g/mouse for ctsk−/− mice). Sv129/C57Bl6 animals (males and females) were started on the HFD at 8–9 weeks of age. At this age, female ctsk−/− exhibited a slightly lower body weight than WT animals. 2-way ANOVA applied to data shown in Figure 2B (top panel) indicate a significant interaction between genotype and time (F = 1.86, df = 11, time X genotype, P<0.05) and a significant (F = 28.03, df = 1, P<0.0001) genotype effect on weight gain, with ctsk−/− female mice showing lower weight gain that WT. Statistical significance for post-tests performed on pairwise comparisons was observable starting from the 9^th^ week of diet (week 9, t = 2.29, P<0.05; week 10, t = 3.3, P<0.05; week 11, t = 4.14 P<0.001; week 12, t = 3.60 P<0.01. Df = 18 in all weeks).

**Table 2:** Effects of HFD on metabolism and weight in ctsk +/+ and ctsk−/− mice.

	Week	Sv129/C57Bl6 females		Sv129/C57Bl6 males		C57Bl6 males	
		+/+	−/−	+/+	−/−	+/+	−/−
Body Weight (g)	0	19.5±0.4 AAA	18.1±0.2	28.3±0.4 A	26.9±0.7	25.7±0.5 AAA	22.4±0.4
	12/13	32.9±1.9	24.9±1.4***	43.2±1.6	39.8±1.1	47.8±1.4	39.1±1.4***
Body Fat (%)	0	13.4±0.7 # AA	11.1±0.5 #	13.1±0.6 #	14.1±0.9 #	-	-
	12/13	33.7±3.8 #	22.1±2.8 # **	32.8±2.7#	32.5±1.0 #	55.6±1.4 §	44.9±2.1***/§
BMD (mg/cm2)	0	47.1±0.4#AAA	51.8±0.5 # ***	50.9±0.6 AA	51.9±1.3	-	-
	12/13	53.4±0.7 #	60.5±1.0 # ***	54.1±0.6	58.6±0.8***	-	-
Non fasting Blood Glucose (mM)	0	7.2±0.2	7.6±0.2	7.8±0.3	8.0±0.4	9.3±0.5	9.6±0.4
	12/13	6.8±0.7	6.1±0.2	7.4±0.5	6.4±0.3	11.7±0.3	11.4±1.0
Triglycerides (mM)	12/13	1.64±0.17	1.59±0.08	1.76±0.11	1.42±0.07*	0.97±0.035	0.79±0.03**
Cholesterols (mM)	12/13	3.73±0.27	3.14±0.12 P = 0.054	5.19±0.24	3.65±0.27**	3.75±0.18	2.41±0.19***
Insulin (ng/ml)	12/13	1.22±0.19	0.61±0.09 P = 0.057	5.96±1.1	3.45±0.6 *	1.45±0.31	1.85±0.27
Leptin (ng/ml)	12/13	32.24±8.73	11.79±4.2*	83.75±14.45	37.24±5.3 *	52.12±3.84	28.3±7.49*

In the “week” column, 12 is referred to sv129Bl6 mice and 13 is referred to C57Bl6 mice.

* p<0.05,

** p<0.01 and

*** p<0.001 compared to the +/+ mice of same sex, same week and same strain (t-test or Bonferroni post-test)

A p<0.05, AA p<0.01, AAA p<0.001 2-way ANOVA.

# Assessed by DEXA,

§ Assessed by MRI

2-way ANOVA applied to data reported in table 2 indicate an overall significant effect of genotype on sv129/C57Bl6 male and female body weight (F = 5.03, df = 1, P<0.05, F = 15.26, df = 1, P<0.001 for males and females respectively): Bonferroni post-tests revealed a significant difference at the end of the treatment in females (week 12) (t = 4.7, df = 18 , P<0.001), but not in males. Indeed ctsk−/− females gained only 51% of the weight gained by WT animals (Figure 2 B, top panel). This result was reflected by the fat content of these animals, as assessed by DEXA, at the end of the study, which was 66% that of WT (2-way ANOVA, F = 8.47, df = 1, P<0.01, post test at week 12, t = 3.41, df = 18, P<0.01). Bone mineral density (BMD, Table 2) was also assessed by DEXA and showed an overall significant increase in ctsk−/− animals as compared with WT both in males and in females (2-way ANOVA, F = 11.32, df = 1, P<0.005, F = 75.23, df = 1 , P<0.0001 for males and females respectively). This is expected given the severely impaired bone resorption determined by ctsk deficiency, which results in the unusually dense trabeculation of the bone marrow spaces, characterizing the osteopetrotic phenotype of these mice [22]. This mechanism seems to completely overcome the increased bone resorption that would result from the relatively low leptin levels [25], [26] observed in the ctsk−/− animals (both on the chow and on the high fat diet) as compared to those of the WT.

A significant resistance to weight gain could be demonstrated in male C57Bl6 ctsk−/− animals (2-way ANOVA, F = 27.4, df = 1, P<0.001, Bonferroni post-test, week 13 of the diet, t = 5.30, df = 18, P< 0.001, Table 2). These animals showed a lower (not significant) body weight at 8 weeks of age, before the HFD was initiated, as compared to wild type controls. This body weight difference became larger as HFD treatment progressed, with C57Bl6 male ctsk−/− mice exhibiting 72% of the final weight gain and 76% of the adipose mass of WT controls (as assessed by MRI) after 13 weeks (Table 2 , MRI data are expressed as 100 x (fat volume/total body volume and Figure 2B, bottom panel). 2-way ANOVA applied to data shown in Figure 2B (bottom panel) indicate a significant interaction between genotype and time (F = 3.05, df = 11, time X genotype, P<0.001) and a significant (F = 11.27, df = 1, P<0.001) genotype effect on weight gain, with ctsk−/− male mice showing lower weight gain that WT. Statistical significance for post-tests performed on pairwise comparisons was observable starting from the 11^th^ week of diet, (week 11, t = 2.26, P<0.05; week 12, t = 3.90, P<0.01; week 13, t = 3.95, P<0.01. Df = 18 in all weeks)

Consistent with the above results, when individual fat depots were dissected at the end of the treatment, C57Bl6 male ctsk−/− mice displayed a significant reduction of epidydimal WAT (t = 3.75, df = 16, P<0.01) perirenal WAT ( t = 2.82, df = 16, P<0.05) and BAT (t = 2.16, df = 15, P<0.05) as compared to WT animals (Figure 2C). Accordingly, histological analysis revealed that adipocyte size was reduced in the ctsk deficient animals (Figure 2D). In addition, at the end of the diet, liver weights were also significantly smaller in C57Bl6 ctsk−/− male mice as compared to WT (1442±64 and 1777±87 mg for ctsk−/− and +/+ mice respectively, t = 2.45, df = 16 P<0.01). Hepatic triglyceride content was lower in ctsk−/− as compared with WT mice (124±34 versus 153±24 mg/g of liver, n = 3), although this difference did not reach statistical significance.

Various metabolic parameters were also assessed at the end of the 12/13-week HFD in C57Bl6 and sv129/C57Bl6 (Table 2). Non-fasting blood glucose levels were not different among the different genotypes in any of the groups, while insulin levels were reduced in sv129/C57Bl6 ctsk−/− females (t = 2.80, df = 18, P<0.05) and males (t = 2.03, df = 22, P = 0.057). Triglycerides were significantly decreased in ctsk−/− male mice of both colonies (t = 2.6, df = 20, P< 0.05, t = 3.97, df = 16, P< 0.01 for sv129/Bl6 and C57Bl6 respectively), but not in sv129/C57Bl6 females, whereas cholesterol was decreased in all groups analyzed (t = 2.06, df = 18, P = 0.05; t = 4.15, df = 22, P<0.05; t = 5.07, df = 16, P<0.001 for respectively sv129/Bl6 females and males and for C57Bl6 males) At the end of the treatment leptin was significantly reduced in all groups of ctsk−/− mice as compared to relative controls (t = 2.11, df = 18, P<0.05; t = 2.80, df = 20, P<0.05; t = 2.91, df = 16, P<0.05 for respectively sv129/Bl6 females and males and for C57Bl6 males).

The same metabolic parameters were also measured in male and female sv129/C57Bl6 ctsk−/− and WT mice fed a regular chow diet over a period of 12 weeks starting at 8–9 weeks of age. Apart from the higher BMD of ctsk−/− animals, no significant differences in body weight were observed for mice with ctsk deficiency compared to WT animals (data not shown).

As noted above, food intake was similar in the two genotypes on either a normal chow or HFD. Moreover, the stool fat content of a further colony of sv129/C57Bl6 female WT and ctsk−/− mice, which were fed a HFD for 12 weeks and which showed a significant difference in weight gain, was not significantly different (data not shown). This result rules out the possibility that the observed differences in fat accumulation and circulating triglycerides were due to differential intestinal absorption or excretion.

The question then rises whether the output of energy was different in the HFD-fed ctsk−/− mice as compared to HFD-fed controls. Since in vivo parameters like O_2 _consumption and body temperature were not measured during the HFD experiment we wanted to retrospectively determine if BAT was differently activated in the two groups of animals. Western blot analysis performed with an antibody directed against mouse uncoupling protein 1, a reliable indicator of BAT activation [27], did not show significant differences between the two genotypes, although an 8% increase was detected in the BAT of C57Bl6 HFD ctsk−/− mice with respect to HFD WT (supplementary data. Figure S1 A and B).

Taken together, the above results suggest that ctsk deficiency confers a partial resistance to the development of morbid obesity on a HFD and that ctsk deficiency protects mice from the onset of dyslipidemia. The effect of the HFD was confined to homozygous ctsk deficient animals as no weight gain, or metabolic effects, were observed with heterozygous animals (data not shown).

### WAT gene expression in young and HFD fed ctsk−/− mice

To examine the molecular basis for the altered adipose mass and lipid profile in the C57Bl6 young and HFD fed male ctsk−/− mice, we measured by real time PCR the expression of genes marking adipocyte terminal differentiation and/or involved in fatty acid metabolism. In young mice, where the adipose tissue is very small and more enriched in undifferentiated cells and stromal vascular fraction (compared to adult animals), we isolated the adipocyte fraction (AF) and pooled samples from 20 animals. In these young mice, gene expression of adipocyte fatty acid-binding protein (aP2), which regulates systemic glucose and lipid metabolism [28], was reduced by 43% in the AF of the ctsk−/− mice as compared to WT animals. At variance, leptin gene expression was not affected. Conversely, in adult ctsk−/− (n = 4) fed a HFD for 12 weeks, we observed a higher expression of aP2 gene (50%) and a lower content of leptin mRNA (−36%) as compared to WT mice. The latter observation is consistent with the lower adipose mass and serum levels of leptin described in HFD fed C57Bl6 ctsk−/− mice (Table 2).

Genes involved in fatty acid metabolism and storage present a consistent picture, in that glycerol phosphate acyltransferase (GPAT), diacyglycerol acetyl transferase (DGAT) and fatty acid synthase (FAS) were all increased in the AF of young ctsk−/− mice (19%, 90% and 45% for GPAT, DGAT and FAS respectively), as well as in adult male ctsk−/− mice fed a HFD for 12 weeks (29%, 18% and 296% for GPAT, DGAT and FAS respectively) compared to WT controls. These data suggest that a pattern of gene activation for lipid synthesis and storage occurs in the WAT of ctsk−/−mice. This is surprising given the reduced fat mass of both young and HFD-fed ctsk−/− mice as compared to controls.

### CPT activity and lipolysis

**Figure 3:**
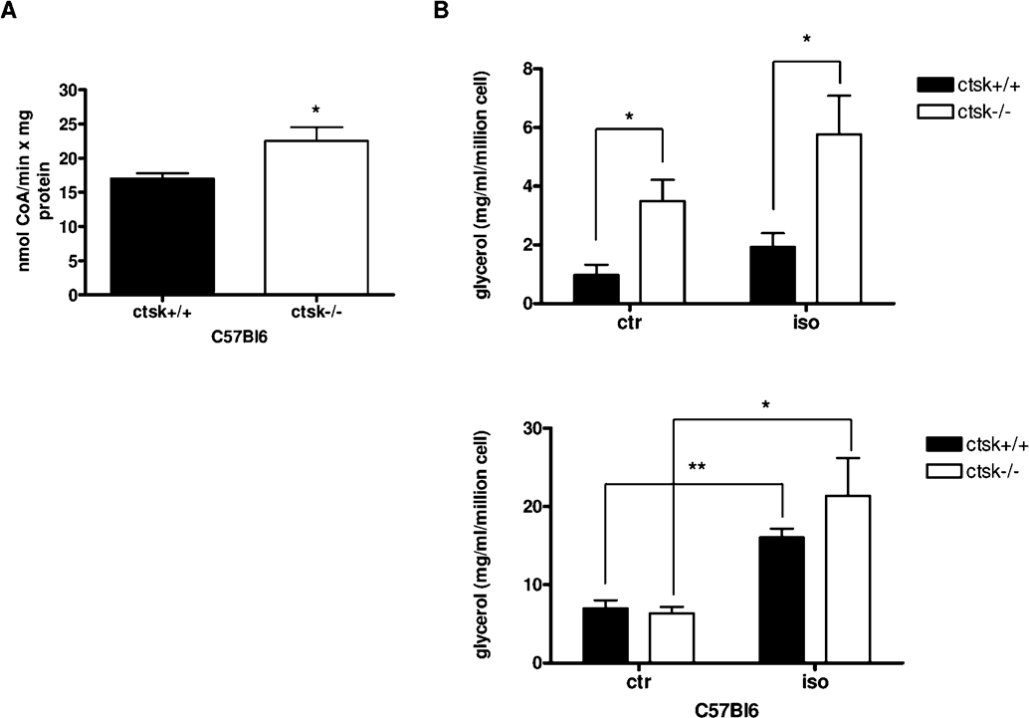
FFA utilization and release in ctsk +/+ and ctsk −/− mice. (**A**) CPT activity of WAT mitochondria from HFD‐fed C67Bl6 WT and ctsk−/− mice. Results are the mean ± SEM for 4 WT and 3 male ctsk−/− mice (40 days‐old) p<0.03 compared to WT mice. (**B**) Glycerol release was measured in adipocytes isolated from the visceral adipose tissue of C57Bl6 WT and ctsk−/− mice maintained in basal medium (ctr), or stimulated for 1 h with 1 µM isoproterenol (iso). Top and bottom panels show respectively the results obtained with young male animals (44 days‐old) and with male animals maintained on HFD for 12 weeks. Results are the mean ± SEM for 4 independent experiments. * p<0.05, ** p<0.01.

Since the above gene expression data point toward a greater storage capacity of ctsk−/− WAT, the leaner phenotype of null mice could be explained if fat cells could also display an enhanced free fatty acid (FFA) oxidation rate. To test this hypothesis, we assessed carnitine palmitoyl transferase-1 (CPT1) activity in isolated mitochondria of ctsk−/− young and HFD-treated (12 weeks) male mice. CPT1 activity was significantly increased (32%, t = 2.89, df = 5, P<0.05) in the WAT of C57Bl6 HFD ctsk−/− mice as compared to WT controls (Figure 3A), whereas there was no difference between young WT and ctsk−/− mice (not shown). This implies a greater mitochondrial delivery of FFA for beta-oxidation in HFD fed ctsk−/− mice and suggests that beta-oxidation exceeds lipid synthesis, thus resulting in a leaner phenotype of HFD-fed ctsk−/− mice as compared to HFD-fed WT animals.

An alternative physiological mechanism leading to a lower adipocyte lipid content could result from differences in FFA release from intracellular stores. Lipolysis was therefore evaluated both under basal conditions and following stimulation with the β3 agonist and lipolytic agent isoproterenol, in adipocytes isolated from young and HFD-treated male C57Bl6 ctsk−/− mice and compared to that of C57Bl6 controls. ANOVA revealed an overall significant genotype effect on lipolysis activity in young (F = 15.6, df = 1, P<0.005) but not in HFD treated animals. Adipocytes from young ctsk−/− mice displayed a significantly increased lipolytic activity (as assessed by Bonferroni post-tests), both in standard assay conditions (3.5-fold, t = 3.15, df = 6 P<0.05) and upon isoproterenol stimulation (3-fold, t = 2.74 , df = 6, P<0.05) (Figure 3B, top panel). Adipocytes from HFD ctsk−/− mice showed a moderate induction (33%) of lipolysis after isoproterenol treatment, although this increase was not statistically significant compared to WT animals (Figure 3B, bottom panel). These data demonstrate that in ctsk deficient animals the impaired capacity for fat storage in adipocytes could be due to a high lipolytic rate in the young and to an increased rate of FFA oxidation in the HFD fed mice. These two mechanisms acting separately, or in combination, could outweigh the effects of the increases in expression of enzymes involved in lipid biosynthesis.

### Adipogenesis in ctsk−/− mouse embryo fibroblasts

**Figure 4:**
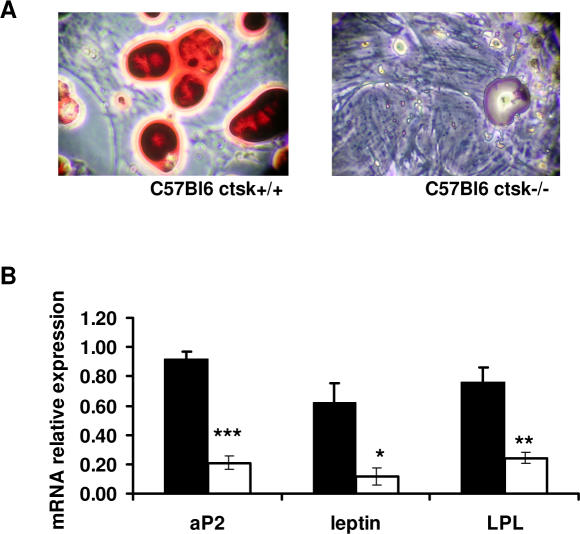
Ctsk deficiency interferes with adipogenesis. (A) Adipocyte differentiation from mouse embryo fibroblasts of C57Bl6 wild type (WT) and ctsk−/− mice (KO). Mouse embryo fibroblasts were prepared 12 days post-coitum and were induced to differentiate in adipocytes. At 10 days after the addition of adipocyte differentiation media we performed oil red O staining (10 X magnification). (B) Quantification of aP2, leptin and LPL mRNA expression after real time PCR in WT (black bars) and ctsk−/− (white bars) MEF. Results are the mean ± SEM for 4 independent experiments. Statistical details for aP2, leptin and LPL are respectively: t = 9.15, t = 2.96 and t = 4.26, df = 5 in all cases. * p<0.05, **p<0.01, ***p<0.001 versus WT.

As shown above, in HFD treated ctsk−/− mice fat depots and adipocyte size are smaller as compared with HFD treated WT. These findings, together with the observation that young ctsk−/− are delayed in the accumulation of fat stores, prompted us to analyze adipocyte differentiation in the absence of ctsk. Mouse embryonic fibroblasts isolated from C57Bl6 ctsk−/− and WT embryos were induced to differentiate, as described in “Methods”. Phase-contrast observations performed on these cells at various time points allowed us to establish that adipogenesis was reduced in cells derived from ctsk−/− mice, as indicated by oil red ‘o staining (Figure 4A). Furthermore, the expression of specific adipocyte markers such as aP2, leptin and lipoprotein lipase was diminished by >60% in ctsk−/− embryonic fibroblasts (t = 9.15, df = 5, P<0.001, t = 2.96, df = 5, P<0.05, t = 4.26, df = 5, P<0.01 for aP2, leptin and LPL respectively) (Figure 4B).

## Discussion

Data presented in this study further strengthen the concept that ctsk is involved in energy balance and more specifically in the homeostasis of adipose mass. Young ctsk−/− mice showed reduced fat mass, as compared to age-matched WT controls. Adult ctsk−/− mice, which do not display overt phenotypic alterations when fed a normal diet, displayed a partial resistance to weight gain and to development of dyslipidemia when challenged with a HFD. The lower body weight was reflected in a diminished fat mass and, consistently, in lower serum leptin concentrations. Variability in weight gain among various colonies of wild-type or ctsk−/− mice may depend on differences in genetic background.

Adipose tissue is an organ with significant plasticity since it is able to undergo rapid dynamic changes. These changes take place during the rapid enlargement of adipose mass in mice soon after weaning and upon metabolic challenge represented, for instance, by the HFD, which forces the animal to store an abnormal excess of energy. In rodents, body fat content at birth is low and WAT is barely detectable [29]. Two major morphologic changes take place and contribute to the development and enlargement of the fat depot in mice. Until the age of 40 days (P40) an intense period of cell adipocyte hyperplasia is observed, whereas the period between P40 and P80 is characterized by hypertrophy. Maximal lipogenic activity, by which adipocytes are filled with triglycerides, peaks at P40 [29]. Interestingly, the most prominent effect on fat mass that we have noticed in ctsk−/− male animals falls between P32 and P56, suggesting that lipogenesis and cell hypertrophy are affected by ctsk deficiency. In females this effect appears earlier, at P21. Further, data obtained on mouse embryonic fibroblasts indicate that ctsk deficiency results in a reduced capacity to undergo adipogenesis, and this further strengthens the concept that this protease is likely involved in the development of adipose tissue, in line with what reported by Xiao et al. [30].

Two different, but not necessarily alternative hypotheses can be formulated to explain the involvement of ctsk in adipogenesis and in situations requiring increased adipose tissue plasticity, such as the postnatal period or feeding with HFD. The first derives from the high capacity of ctsk to digest important components of the extracellular matrix, such as type I collagen and elastin. Because of this activity, ctsk has been implicated in diseases involving bone and cartilage destruction [31]–[33]. Ctsk has also been detected in breast and prostate cancer where its role seems to be associated with the invasive potential of these tumours, in addition to matrix degradation [34], [35]. Enlargement of the adipose mass, such as that seen in the post-weaning period and upon HFD feeding, involves massive remodelling caused by increased cell size and number and by new vessel formation. Thus, the activity of ctsk could facilitate this process to which other proteases also participate. Therefore, in the long term and in a static situation (such as in the adult mouse) an effect of ctsk deficiency may not be observed since its function may be compensated by the upregulation of other proteases. Metalloproteases (MMP-2 and MMP 9) are present in WAT, where they play a role in adipogenesis [15] and recent work by Taleb et al. [12] shows that the cysteine protease cathepsin S is expressed in WAT where it promotes adipogenesis potentially via the degradation of fibronectin.

The second, more speculative, hypothesis figures a role for ctsk not only in the environment surrounding the adipocyte, but also in the activation of pathways affecting adipocyte physiology and metabolism. It is difficult to explain such effects for a lysosomal and secreted protein, known to act on the extracellular matrix. Unfortunately, antibodies directed against ctsk which are reported to work well in other tissues, give a very poor and often non-specific signal in the WAT, and for this reason, our attempts to better define the localization of ctsk within adipose tissue have so far been unsuccessful. However, recent findings indicate that other cathepsins can change their localization depending on cellular conditions. For instance, cathepsin L (ctsl) isoforms devoid of the signal peptide, can localize to the nucleus and process the transcription factor CDP/Cux involved in the G1/S transition [36]. Such isoforms are translated from ATGs located downstream of the methionine codon 1. Although the amino acid sequences of ctsk and ctsl present only 45% homology, the internal translation start sites present in ctsl (Met 77 and Met 83) are perfectly conserved in ctsk. Indeed, Goulet and coworkers report that ctsk is capable of cleaving CDP/Cux, although to a lesser extent [36]. Based on these observations it is possible that ctsk is able to localize in subcellular compartments other than the lysosome and participate in the activation of metabolic pathways.

**Figure 5:**
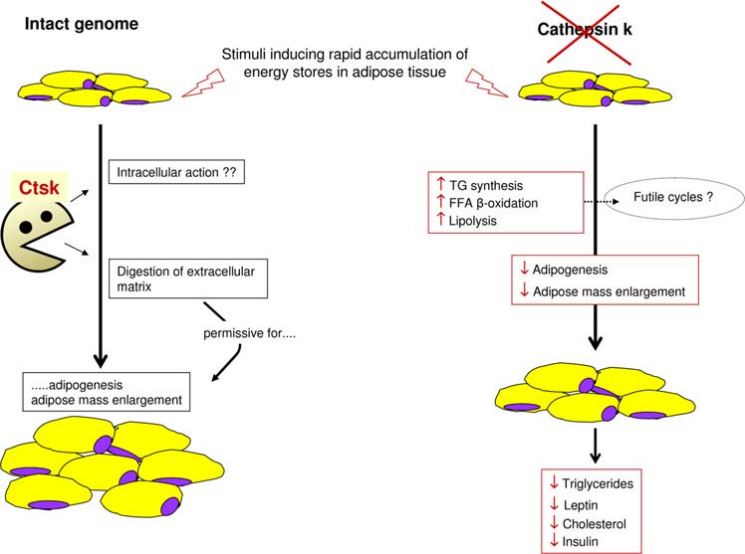
Hypothetical scheme representing how ctsk ablation in mice may result in partial resistance to increase adipose mass in conditions requiring rapid accumulation of fat stores. In wt mice (intact genome, left side of the panel) ctsk participates to WAT extra-cellular matrix remodelling, thus facilitating the enlargement of the adipose mass, which implies adipocyte size increase and de novo differentiation of pre-adipocytes. Mechanisms involving an intracellular action of ctsk are also hypothesized. In ctsk deficient animals (right side of the panel) stimuli directed to increase the adipose mass are partially blunted and the extra caloric intake is partly utilized in a futile cycle, consisting of increased synthesis of triglycerides, increased triglycerides hydrolysis and increased β-oxidation of FFA.

The reduced capacity of ctsk−/− mice to accumulate fat mass in spite of an equal caloric intake suggests that the absence of ctsk is associated with increased energy expenditure, which might be due to increased BAT activation. Yet, we could not demonstrate a significant increase of UCP1 content in BAT from ctsk−/− mice as compared with controls. This does not rule out an involvement of BAT in increased thermogenesis of ctsk−/− mice but other, possibly additional, mechanisms acting in other tissues should be considered. Indeed, we found that the reduced capacity of ctsk−/− mice to accumulate fat mass is not due to decreased expression of lipogenic enzymes but to an increased turn-over of free fatty acids. Indeed, on one hand our gene expression studies in WAT point toward a pattern of induction in a number of genes involved in fatty acid synthesis (increased FAS) and esterification (moderately increased GPAT and DGAT), which should favour and not hinder TG storage. On the other hand, a highly enhanced rate of lipolysis and increased CPT-1 activity were detected in the WAT of young and HFD-fed adult ctsk−/− mice, respectively. Such metabolic features suggest that adipocytes of ctsk−/− mice have an increased capacity to release and/or oxidize fatty acids, which makes them less prone to TG storage during early post-natal development or upon increased lipid availability, despite enhanced expression of lipogenic enzymes. It is likely that in ctsk-deficient animals, the turnover of FFA is accelerated because of enhanced lipolysis and an increased rate of the futile TG-FFA cycle (particularly in young mice), as well as an enhanced FFA utilization for beta-oxidation (particularly in HFD-fed adult mice). These changes ultimately result in the less efficient storage of triglycerides. The increased rate of FFA recycling, which in fasting rodents and humans accounts for 30–40% of WAT FFA disposal [37], is reminiscent of a hyperthyroid phenotype, which however can be ruled out based on the slight hypothyroidism of the ctsk−/− mice [5]. The similar, if not higher, degree of BAT activation observed in HFD-fed ctsk−/− mice, despite their lower body fat content, suggests that energy expenditure is also probably slightly increased in this model. An hypothetical model of how cathepsin k deficiency impacts on accumulation of fat mass is represented in figure 5.

Overall our data indicate that ctsk ablation in mice results in a reduced body fat content under conditions requiring a fast accumulation of fat stores, namely, the post weaning period and the exposure to a high fat diet. This observation emphasizes the similarity of these two conditions and indicates ctsk as a permissive factor for WAT “plasticity”, intended as capacity to rapidly accumulate energy stores. This may have important implications to understand the onset of obesity in the very young and the relationship between childhood and adulthood obesity.

The reduced body fat content observed in ctsk−/− mice can be partly explained by an increased release and/or utilization of free fatty acids and potentially by an augmented ratio of lipolysis/lipogenesis. Further, our results demonstrate that under a HFD, ctsk deficiency confers a partial resistance to the development of dyslipidemia. Unfortunately current human ctsk K inhibitors are neither potent, nor selective against the rodent enzymes, therefore precluding the testing of this hypothesis pharmacologically in mice or rats. However, ctsk inhibitors are presently under evaluation in clinical trials for the treatment of osteoporosis, osteoarthritis and metastatic bone disease [38]. This will enable the metabolic effects observed in mice to be more fully explored clinically in humans.

## Methods

### Experimental animals

The strategy used to target the cathepsin K locus and generate ctsk null mice was as previously described [22]. These animals were originally generated on a C57Bl6 x sv129 background. Two colonies of mice were used in this study. Animals used both for the characterization of the adult and young phenotype and in the high fat diet experiment were backcrossed for 8 generations onto a C57Bl6 background. An additional group of animals, used only in the high fat diet experiment, were on the mixed sv129/C57Bl6 background. All studies comparing ctsk−/− and WT mice used littermate animals. We used the parental wild type inbred strain as controls. Mice (0–12 months of age) were housed in a pathogen free barrier facility (12-h light/12-h dark cycle). Mice were placed on either a normal chow (2018 Teklad global diet, 18.9% protein, 5.7% fat and 57.3 % carbohydrate, Harlan Teklad) or on high fat high carbohydrate diet for 12 weeks (Diet F3282, 19% protein, 35% fat and 35 % carbohydrate, Bioserve Frenchtown, New Jersey). During the HFD treatment, food intake and body weight were measured weekly. Animals were killed by CO_2_ asphyxiation or cervical dislocation. Gonadal and perirenal fat pads, brown adipose tissue and liver were dissected, weighed and then promptly frozen in liquid nitrogen for biochemical and molecular studies. Blood was withdrawn from euthanized animals by cardiac puncture. All animal protocols were approved by the local ethical committees. The experimental protocols followed the Principles of Laboratory Animal Care (US NH publication N° 83–85, revised 1985).

### Blood collection

Unless otherwise specified, metabolic parameters were assessed in blood samples obtained from conscious non-fasting animals between 10 and 11 a.m. Blood samples were taken from the tail vein using EDTA coated tubes and centrifuged in a refrigerated microfuge. Plasma was collected and stored at −20°C for subsequent assays.

### Intraperitoneal glucose tolerance test

Glucose (1.5 g/kg) was given as a 16.5% solution to conscious non-fasting animals. Blood samples were collected sequentially from the tail vein before and 15, 60, 120 and 210 min after glucose injection.

### Assays

Plasma glucose was measured by a glucometer (Precision QID, MediSense) or by the glucose oxidase method using commercial kits (Glu-cinet, Sclavo Diagnostics International, Siena, Italy). Plasma insulin was measured by radioimmunoassay (RIA) according to Herbert et al. [39], using insulin antibody and ^125^I-labeled insulin from Linco (Linco Research, Inc., St. Charles, MO, USA). FFA concentrations were determined by the Wako NEFA C test (Wako Chemicals GmbH, Neuss, Germany). Plasma TG and cholesterol levels were assayed using commercially available kits (Vitros or Chema Diagnostica, Iesi, Italy). Plasma leptin was measured by radioimmunoassay, using a Linco RIA kit, specific for mouse leptin (Linco Research, Inc., St. Charles, MO, USA). Alternatively mouse insulin and leptin were determined simultaneously by electrochemiluminescence (Meso Scale Discovery).

### Isolation of total RNA and real time PCR

Total RNA was isolated from frozen tissues or from cells with Tripure (Roche Molecular Biochemicals) and its integrity was evaluated on a formaldehyde denaturing agarose gel. RNA was treated with Rnase-free Dnase (Roche Molecular Biochemicals) to remove any contaminating genomic DNA. First-strand cDNA synthesis was performed using oligo hexamers (Pharmacia) (see [19] for detailed protocols).

Taq-Man quantitative PCR (50°C for 2 min, 95°C for 10 min, followed by 40 cycles of 95°C for 15 s 60°c for for 1 min) to amplify samples for aP2, glycerol phosphate acyltransferase (GPAT), diacyglycerol acetyl transferase (DGAT), fatty acid synthase (FAS), lipoprotein lipase (LPL) and leptin. The relative abundance of mRNAs was calculated with Tata Binding Protein mRNA as the invariant control. The primers used were all purchased from Applied Biosystems.

### Preparation of isolated adipocytes

Isolated fat cells were obtained with a modification to the method of Rodbell [40]. Briefly, visceral adipose tissue was dissected, rapidly washed in PBS, then minced and digested for 1 h with shaking at 37°C with Collagenase A (20 mg/ml from Clostridium histolyticum, SIGMA # 103578) in Krebs Ringer Bicarbonate buffer (KRBH-buffer), containing 10mM NaHCO_3_,30 mM Hepes and 1% BSA(Sigma) at pH 7.4.

1 ml of collagenase solution was added to 4 g of fat pad in KRBH-buffer. Fat cells were filtered through nylon mesh (250 µm) and washed twice with the same incubation buffer. After the final wash the adipocytes were suspended in Dulbecco ‘s modified Eagle’s medium (DMEM) supplemented with 10% FCS (Gibco), 200U/ml penicillin and 50 µg/ml streptomycin.

### Measurement of lipolysis

Measurement of lipolytic activity was performed by incubating isolated adipocytes (10^6^ cells) in 2 ml of KRBH- buffer for 4 h at 37°C with 5% CO_2_ in the absence and in the presence of 1 µM isoproterenol. After 4 h of incubation 1ml of the incubation medium was removed and acidified with 100 µl of 30% trichloroacetic acid. The mixture was vigorously shaken and then centrifuged at 3000g for 10 min at 4°C. A volume of 700 µl of surnatant was collected and neutralized with 80 µl of 10% KO and assayed for glycerol content (GPO Trinder, SIGMA)

### Protein preparation

Adipose tissue was homogenized in a lysis buffer containing 20 mM Tris (pH 7.5), 150 mM NaCl, 10% glycerol, 1% Triton X-100, 10 mM EDTA, and 1 mM phenylmethylsulfonyl fluoride. The homogenate was then centrifuged at 3000 rpm for 30 min at 4°C. The top and bottom layers were discarded, and the intermediate phase was collected and assayed for the protein concentration by using the the Bradford Reagent (Bio-Rad).

### Immunoblotting

Proteins were separated on 12% SDS-PAGE and transferred onto a nitrocellulose filter by electroblotting. Filters were saturated with a solution of 5% dry milk in Tris-buffered saline (TBS) and incubated with a polyclonal primary antibody anti-mouse uncoupling protein-1 (UCP-1) raised in rabbit (Calbiochem, cat. no 662045), at a 1:2000 dilution in TBS 0.1% Tween with 2% dry milk. After three washes with TBS, the filter was incubated for 1 h in the anti-rabbit secondary antibody (horseradish peroxidase conjugated), diluted 1:3000 in TBS 0.1% Tween 2% dry milk. Specific protein expression was visualized using a chemiluminescent assay (Amersham Pharmacia/Biotech, Piscataway, NJ), after exposure to x-ray film for 1–5 min. β-tubulin (TO198, Sigma, St. Louis MI), at a dilution of 1:600, was used as an internal control to verify equal loading of the protein. The intensity of the bands was quantified using Quantity One 1-D Analysis software (Biorad, Hercules CA).

### Isolation of mitochondria and CPT assay

Adipose tissue mitochondria were isolated by differential centrifugation. Freshly isolated tissue was minced on ice and then homogenized with a Potter-Elvehjem in homogenization buffer 1 (220 mM mannitol, 70 mM sucrose, 20 mM Tris-Hcl, 1 mM EDTA) 1:6 (w/v). The homogenate was centrifuged at 22.500 g for 25 min at 4°C and, after removing of the fat layer, the pellet was resuspended in the same buffer. This suspension was centrifuged at 700 g for 10 min at 4°C. Pellet was discarded and supernatant was then recentrifuged at 15000 g for 30 min at 4°C to pellet mitochondria. Mitochondria were immediately used for CPT assay or stored at – 80°C.

Carnitine palmitoyl transferase activity was assayed spectrophotometrically following carnitine-dependent CoA liberation in presence of palmitoyl-CoA and DTNB at 412 nM, according to the method of Bieber et al. [41] with some modifications. The reaction buffer was: 116 mM Tris-HCl, 0.09% Triton, 1.1 mM EDTA, 35 µM palmitoyl-CoA, 0.12 mM DTNB and 3.3 mM carnitine in a final volume of 0.9 mL. Assay buffer without mitochondria was used as a blank. Mitochondrial proteins (100 µg) were then brought to a final concentration of 1 µg/µL in buffer 1 containing 0.1% Triton, added to the assay buffer, incubated 30 s and then the reaction was started. Change in absorbance was monitored for 3 min and CPT activity was expressed as nmol CoA/min x mg protein.

### MRI

Magnetic resonance images (MRI) were acquired using a Biospec Tomograph (Bruker, Germany) operating at 4.7 T. Mice were anaesthetized through Avertin (10 µl/gram of body weight) and placed in the supine position in a 7.2 i.d. bird cage volume coil. This large coil was chosen in order to achieve a uniform excitation of the whole mice body. Transversal spin echo images were acquired using the following parameters: TR = 530 ms; TE = 10.2 ms; Slice thickness = 2 mm; FOV = 6×3 cm^2^, Matrix size = 256×128 (in plane space resolution = 234×234 m^2^ ). Three packages of 15 contiguous slices were acquired in order to cover the whole mice body. MRI data were analyzed using the software Paravision (Bruker, Germany). A single investigator, blinded to the experimental groups of mice, analyzed the images. Fat tissue appears strongly hyperintense compared to the remaining body organs. Pixels attributable to fat could be segmented in each slices by a simple threshold algorithm: pixels having signal intensity higher then the threshold value were attributed to fat tissue. After thresholding each slice was visually inspected in order to verify that the algorithm correctly selected only fat pixels. A similar algorithm based on a threshold value was used for the quantification of total body volume. In each slice the total fat and body volumes were calculated according to:
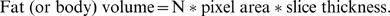
Where N represents the number of pixels attributed to fat or to body, respectively.

### Body Fat Analysis by DEXA

Dual Energy X-ray Absorption (DEXA, PIXImus, Lunar Corp) was used to evaluate percent body fat content in mice from sv129/C57Bl6. Animals were anesthetized with mixture of ketamine and xylazine, placed on the scanning apparatus, and bone mineral density (BMD) and percent body fat determined. Animals were kept in a warm environment under a heating lamp until fully recovered.

### Assessment of liver triglyceride (TG) content

Liver triglyceride content was determined by extracting total lipids from liver samples with chloroform:methanol (2:1 vol/vol) as described by Folch et al. [42], separating the chloroform and methanol–water phases twice, discarding the upper phase and finally evaporating the lower phase under N_2_. For the assay, samples were resuspended in 100 µl chloroform; 30 µl were quickly transferred to glass tubes in duplicate and air dried. After addition of 50 µl of bidistilled water they were vortexed and incubated at 37°C in a shaking water bath for 10 min. One milliliter of GPO Trinder reagent (Sigma-Aldrich) was added to the tubes, which were then gently mixed and incubated at 37°C for 5 min. The absorbance of duplicate samples was read at 540 nm in a spectrophotometer. The range of linearity of this method based on GPO Trinder reagent is up to 1000 mg/dl and the detection limit is 1.0 mg/dl.

### Histological analysis

Animals were anesthesized with Avertin and then perfused using 4% paraformaldehyde. Epidydimal adipose tissue samples were then post-fixed using 4% paraformaldehyde for 12-24 h and embedded in paraffin. Five micrometers sections were mounted on charged glass slides and then stained with hematoxilin-eosin.

### MEF isolation and treatments

Primary MEFs were isolated from 12 days post-coitum mouse embryos [43]. Embryos were surgically removed, and separated from maternal tissue and the yolk sack. The bodies were minced finely and then incubated in a solution of trypsin:EDTA (0.05% trypsin; Sigma; 1 mM EDTA, glucose, 1x PBS) with shaking at 37°C for 15–30 min. The solution was allowed to settle for 2 min, and the surnatant was centrifuged for 3 min at 1200 rpm. The resulting pellet was resuspended in culture medium and cells were plated at 10^4 ^cells/cm^2 ^. Attached cells then constituted passage 1.

All primary MEFs were maintained in Dulbecco's Modified Eagle Medium (DMEM, Gibco) supplemented with 10% fetal bovine serum (FBS, Gibco), 100 units of penicillin and 100 µg/ml streptomycin. Cultured cells were maintained at 37°C in a humidified 5% CO_2 _atmosphere. For adipocyte differentiation MEFs were plated on 35 mm dishes and at two days post-confluence the medium was supplemented with 5 µM dexamethasone (Sigma), 0.2 mM isobutylmethylxanthine (Sigma) 1 µM BRL-49653 (kindly provided by Glaxo Smithkline) and 10 µg/ml insulin(Sigma) for 3 days followed by supplementation with only insulin and BRL-49653 from day 3.

### Statistical analysis

The number of mice in each experimental group is indicated in the figure legends. All values are expressed as means±SEM. Pairwise comparisons of quantitative phenotypes between mice of different genotypes (e.g. obese ctsk−/− versus obese ctsk +/+) were assessed by 2-tailed Student's t-test. When more than 2 groups were analyzed, 2-way ANOVA followed by Bonferroni post-test for selected comparisons (e.g young ctsk +/+ versus young ctsk−/−) was used. Spearman linear regression analysis was performed to evaluate association between variables. A significance limit of P<0.05 was set.

## Supporting Information

Figure S1 A. Representative immunoblotting showing UCP-1 and β-tubulin protein expression in mouse BAT of C57Bl6 ctsk +/+ and C57Bl6 ctsk −/− mice. Total protein extracts from mouse BAT(10ug) were separated by SDS-PAGE and transferred onto nitrocellulose. Blots were respectively probed with primary antibodies against mouse UCP-1 and β-tubulin and horseradish peroxidase-conjugated secondary antibody. B. Bar graph showing the relative expression of UCP-1 in the BAT of HFD ctsk−/− and +/+ mice. Quantity One 1-D Analysis software was employed to quantify band intensity. Levels of UCP-1 were corrected for by the intensity of the corresponding β-tubulin band.
(3.97 MB TIF)
