# Supplementary figures and images for: Cathepsin K Null Mice Show Reduced Adiposity during the Rapid Accumulation of Fat Stores
Source: PLoS One. 2007 Aug 1;2(8):e683. doi: 10.1371/journal.pone.0000683 (PMC1925145; doi:10.1371/journal.pone.0000683)

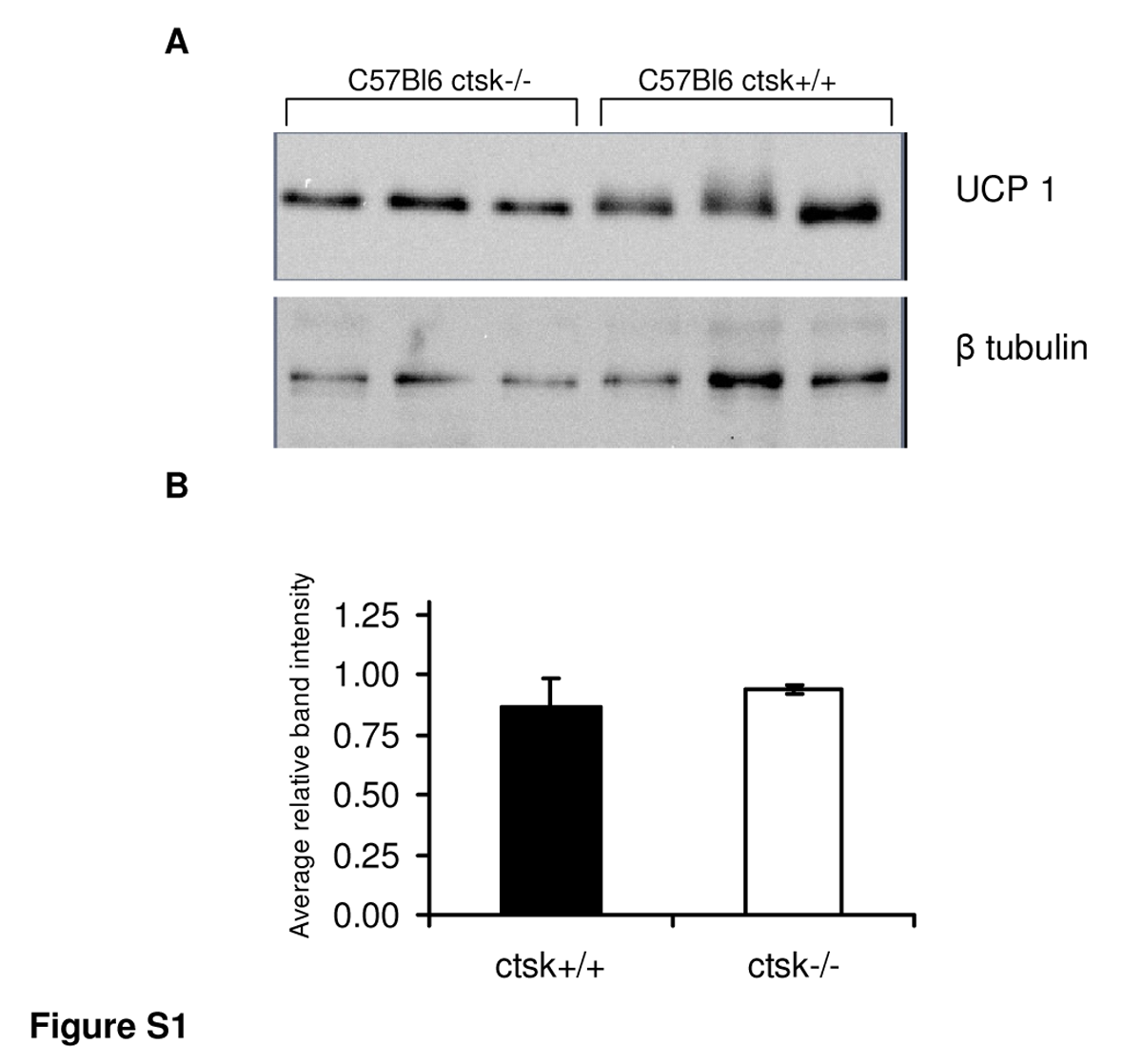

Supplement: Figure S1 — A. Representative immunoblotting showing UCP-1 and β-tubulin protein expression in mouse BAT of C57Bl6 ctsk +/+ and C57Bl6 ctsk −/− mice. Total protein extracts from mouse BAT(10ug) were separated by SDS-PAGE and transferred onto nitrocellulose. Blots were respectively probed with primary antibodies against mouse UCP-1 and β-tubulin and horseradish peroxidase-conjugated secondary antibody. B. Bar graph showing the relative expression of UCP-1 in the BAT of HFD ctsk−/− and +/+ mice. Quantity One 1-D Analysis software was employed to quantify band intensity. Levels of UCP-1 were corrected for by the intensity of the corresponding β-tubulin band. (3.97 MB TIF) [file pone.0000683.s001.tif]
